# Kisspeptin Activates *Ankrd* 26 Gene Expression in Migrating Embryonic GnRH Neurons

**DOI:** 10.3389/fendo.2016.00015

**Published:** 2016-03-01

**Authors:** Tomoko Soga, Wei Ling Lim, Alan Soo-Beng Khoo, Ishwar S. Parhar

**Affiliations:** ^1^Brain Research Institute, School of Medicine and Health Sciences, Monash University Malaysia, Bandar Sunway, Malaysia; ^2^Molecular Pathology Unit, Cancer Research Centre, Institute for Medical Research, Kuala Lumpur, Malaysia

**Keywords:** metastasis, cancer, cytoskeleton, preoptic area, reproduction, GPR54

## Abstract

Kisspeptin, a newly discovered neuropeptide, regulates gonadotropin-releasing hormone (GnRH). Kisspeptins are a large RF-amide family of peptides. The kisspeptin coded by KiSS-1 gene is a 145-amino acid protein that is cleaved to C-terminal peptide kisspeptin-10. G-protein-coupled receptor 54 (GPR54) has been identified as a kisspeptin receptor, and it is expressed in GnRH neurons and in a variety of cancer cells. In this study, enhanced green fluorescent protein (EGFP) labeled GnRH cells with migratory properties, which express GPR54, served as a model to study the effects of kisspeptin on cell migration. We monitored EGFP–GnRH neuronal migration in brain slide culture of embryonic day 14 transgenic rat by live cell imaging system and studied the effects of kisspeptin-10 (1 nM) treatment for 36 h on GnRH migration. Furthermore, to determine kisspeptin-induced molecular pathways related with apoptosis and cytoskeletal changes during neuronal migration, we studied the expression levels of candidate genes in laser-captured EGFP–GnRH neurons by real-time PCR. We found that there was no change in the expression level of genes related to cell proliferation and apoptosis. The expression of ankyrin repeat domain-containing protein (ankrd) 26 in EGFP–GnRH neurons was upregulated by the exposure to kisspeptin. These studies suggest that *ankrd 26* gene plays an unidentified role in regulating neuronal movement mediated by kisspeptin–GPR54 signaling, which could be a potential pathway to suppress cell migration.

## Introduction

Kisspeptin is a newly discovered neuropeptide that controls the onset of puberty and stimulates gonadotropin-releasing hormone (GnRH) release from the preoptic area (POA). Kisspeptin encoded by KiSS-1 gene is a 145-amino acid peptide. The primary translation products are shorter, biologically active cleavage neuropeptides, kisspeptin-54, -14, -13, -10 amino acid residues, which are endogenous ligands for G-protein-coupled receptor 54 (GPR54) (1–3). Kisspeptin–GPR54 signaling is coupled with Gq*/*11 (1, 2). Intracellular kisspeptin–GPR54 pathway activates Ca^2+^-dependent signals, such as Ca^2+^ mobilization and phosphorylated by mitogen-activated protein kinases (1). Kisspeptin synthesizing neurons are localized in the hypothalamus, but expression of kisspeptin and GPR54 is also found in various peripheral tissue, such as the placenta, pancreas, the testes (3–5), and notably, GPR54 is expressed in metastatic human cancer cell lines (6).

Reproductive neuropeptide, GnRH stimulates the secretion of pituitary gonadotropins and activates release of gonadal hormones to control reproductive activity. Interestingly, GnRH neurons have a unique developmental process. Embryonic GnRH neurons have high degree of motility; they originate from the olfactory placodes and migrate into the POA, their final residing place in the brain of all vertebrates (7–10). In rats, GnRH neurons actively start to migrate from the olfactory placodes on embryonic day 13.5 (E13.5) (11) and reach their final position in the POA on E18−19 (12). Approximately 1500 GnRH neurons are observed during early developmental stages, and these remain unchanged in numbers in the POA during adult stages (11, 12). Abnormal development of GnRH neurons, such as failed embryonic migration, altered numbers or positioning in the POA causes hypogonadotropic hypogonadism and reproductive dysfunction (13, 14).

Although kisspeptin−GPR54 signaling in GnRH neurons is well demonstrated as a key component for the onset of puberty (15–18), GnRH neurons express GPR54 before pubertal onset and kisspeptin–GPR54 system are established in embryonic mice brain (19, 20). In addition, recent study suggests that neuronal connection of kisspeptin neurons in the arcuate nucleus and a specific subgroup of GnRH neurons are established during embryonic stage in mice brain (21). An important role in controlling reproduction is emblematic of kisspeptin’s biological functions (22); however, this peptide is multifunctional (5, 23). The peptide has been shown to regulate cognitive functions, such as anxiety, learning, and memory (24–26), and as a metastasis suppressor (Metastin = Kisspeptin 54) (2). There are no studies describing the function of kisspeptin–GPR54 cellular signaling in normal migrating GnRH cells in the developing brain. However, an earlier study had suggested the possibility of GPR54 as a “stop molecule” for GnRH neuronal migration in the brain of a teleost fish (27).

Several studies have shown that kisspeptin is involved in cancer cell invasion (28–30), cell migration (2–4, 6), cell cycle arrest, and induction of cancer cell apoptosis (31). The cellular mechanisms of kisspeptin’s inhibitory effect on cell invasion, migration, cell cycle arrest, or induction of apoptosis have been suggested to be *via* altered cell motility and adhesiveness (32).

As accumulating evidences of kisspeptin neurons and GPR54 expression in early developmental stage, it is possible that kisspeptin–GRP54 signaling may be functionally involved in embryonic migrating GnRH neurons long before puberty and reproductive maturate stage. This study was designed to determine whether kisspeptin-10 could influence embryonic GnRH neuronal migration, which could be important in the understanding of the mechanisms of neuronal migration. Here, we report that kisspeptin-10 activates *ankrd26* gene expression in migrating embryonic GnRH neurons and inhibits their ability to migrate.

## Materials and Methods

### Animals

Transgenic Wistar rats expressing the enhanced green fluorescent protein (EGFP) under control of the rat GnRH promoter (33) were used to enable the direct visualization of GnRH neurons. The rats were housed as groups (2−3/cage) under a 12-h light cycle (lights on from 12:00 a.m. to 12:00 p.m.) and controlled temperature (22.0 ± 1.0°C) in the Specific Pathogen Free animal facility of the Brain Research Institute, Monash University Malaysia. Food and water were available *ad libitum*. All procedures were carried out in accordance to the Guidelines for the Care and Use of Animals by Monash University (SOBSB/MY/2007/29, MARP/2011/064).

### Dissection and Brain Slice Preparation

Adult female rats were time-bred, with the presence of spermatozoa in vaginal smear designated as on day 0 of gestation (E0). Pregnant female rats on E14 were anesthetized with an intraperitoneal injection of ketamine xylazine (4.5 mg/kg/body weight). The embryos were immediately removed from the uterus and placed on glass dish with cold 1× Krebs buffer (126 mM NaCl, 2.5 mM KCl, 2.5 mM CaCl_2_ 1.2 mM NaH_2_PO_4_, 1.2 mM MgCl_2_, 25 mM NaHCO_3_, and 11 mM d-glucose, pH 7.2, sterile filtered). The embryonic brains were dissected in the cold Krebs buffer and embedded in 6.0% low gelling temperature agarose (Type VII-A, Sigma, St Louis, MO, USA) to harden on ice. The embedded brains were trimmed, glued with cyanoacrlylate (Loctite 404, Henkel Corporation, Rocky Hill, CT, USA) to the stage of vibratome (Vibratome 3000 Sectioning System, St. Louis, MO, USA), and sagittally sectioned at 300 μm thickness in cold Krebs buffer. The slices were collected into glass-bottom dish filled with Krebs buffer supplemented with HEPES buffer solution (GIBCO-Invitrogen, Carlsbad, CA, USA), gentamicin reagent solution (GIBCO-Invitrogen), and antibiotic-antimycotic solution (GIBCO-Invitrogen). All sections were then incubated in medium [DMEM/F12 Phenol Red Free (GIBCO-Invitrogen), B-27 serum-free supplement (GIBCO-Invitrogen), l-glutamate, antibiotic–antimycotic solution, l-glutamine (Sigma), and d-glucose (Sigma)]. The sections were viewed under inverted fluorescence microscope (Nikon TE2000, Japan) for selection of brain slices containing EGFP–GnRH neurons.

### Embryonic Brain Slice Culture

The embryonic brain slice selected for live cell imaging were adhered to the glass-bottom WillCo-dish (WillCo Wells BV, Amsterdam, Netherlands) using chicken plasma (10 μl; Sigma) with thrombin (25 μl; Sigma) (34) followed by 1.5 h incubation at 37°C. After incubation, 1 ml of culture medium [serum-free Neurobasal medium (GIBCO-Invitrogen) supplemented with B27, l-glutamine, antibiotic–antimycotic solution, and d-glucose] was added into the dish. Slices were maintained in culture medium at 37°C, humidified with 5% CO_2_ for 3 h to allow the slices to adhere to the glass coverslip and acclimatize to the culture condition (35) before imaging. For the exposure to kisspeptin, E14 embryonic slices were maintained in culture medium for 12-h post plating and kisspeptin-10 [1 nM, KiSS-1 (110–119) Amide, cat# 048-65, Phoenix Pharmaceuticals, Inc., Burlingame, CA, USA] was added twice at 12 h interval for static incubation. Imaging of embryonic EGFP–GnRH neurons was continued for an additional 12 h. Procedures for the brain slice culture and medium preparation were adapted from Bless et al. (36), with slight modifications to accommodate to the live cell imaging system.

### Live Cell Imaging of Embryonic EGFP–GnRH Neurons

The dish containing the attached E14 brain slice with culture medium was transferred into the chamber attached to the stage of Leica AF6000LX Live Cell Imaging System. The chamber temperature was maintained at 37°C, humidified with 5% CO_2_ (PeCon GmbH, Erbach, Germany). EGFP–GnRH neurons were visualized and captured under the 20× objective lens of the inverted fluorescence microscope. Time-lapse image analysis of the EGFP–GnRH neurons from E14 (*n* = 6–13 cells/time point) were expressed as the total distance traveled (μm/12 h) and the average rate of movement (μm/h) at 12 h interval for 36 h duration.

The exposure time, gain, and intensity parameters were kept constant during imaging A *z*-series of 25 images through the brain slice containing EGFP–GnRH neurons were collected every 4–5 μm interval (~100−125 μm), at 5 min interval for total 36 h of video microscopy. The *z*-series of images collected were processed using the analysis software (Leica LAS AF) into projected stacks, and video images were generated for the analysis of GnRH neuronal migration. The live cell image of GnRH migration was captured wildly throughout the olfactory bulbs, junction area and ventral forebrain in the sagittal slice culture. All data of GnRH migration were averaged in each anatomical area in E14 developmental stage.

### Video Analysis

Due to the long-term observation of GnRH neurons, images of the EGFP–GnRH neurons were collected every 5 min interval for 9, 12, or 15 h each session until 36 h of recording were completed. The video images were analyzed using Image Pro-Plus version 6.0 (Media Cybernetics Incorporation, Bethesda, MD, USA) to track movement of EGFP–GnRH neurons for absolute distance and velocity across the recording period. The absolute distance is defined as the distance traveled by the neurons captured at each frame in the video image sequence. The velocity generated by the software was calculated by dividing the duration of each frame (5 min) in the video sequence by the absolute distance traveled. Data for the embryonic EGFP–GnRH neuron migration were presented as total distance traveled and the average rate of neuronal movement at 12 h interval. Data from the video images of EGFP–GnRH neurons that showed notable tissue movement were discarded.

### Real-Time PCR for Laser-Captured Single EGFP–GnRH Cell

After kisspeptin-10 treatment for 24 h (twice at 12 h interval), E14 embryonic brain slices were washed in 0.1M phosphate buffered saline (PBS) and fixed in 4% paraformaldehyde (PFA) [buffered in 0.1M phosphate buffer (PB)]. The brain slices were re-cut in 14 μm, were pasted on APS-coated slides (Fisher Scientific), and were washed in diethylpyrocarbonate-treated water for 20 s (5 s dips). The protocol used for laser capture single EGFP–GnRH and real-time PCR was as described previously (37). The tissues were dehydrated in an ethanol series (75% V/V, 95% V/V, and 100%, 10 s each) and air dried. GnRH cells were dissected using a UV laser component of Arcturus Laser Capture Microdissection System (Arcturus, Mountain View, CA, USA) powered at 31%, slow speed cutting. The dissected cells (20–25 cells/animal) were pooled and collected into a microcentrifuge tube containing 200 μl of chilled TBS using a micropipette attached to a micromanipulator (Narishige, Tokyo, Japan). The collected cells were precipitated by centrifugation at 3,000 rpm for 20 min at 4°C and the supernatant was discarded. The harvested cells were resuspended in 12.9 μl of lysis buffer solution [7.9 μl of DEPC-treated water, 1.2 μl of 10× first strand buffer, 1.2 μl of 40% NP40, 0.6 μl of Proteinase K (1 mg/ml), and 2 μl of glycogen (5 ng/ml)] and incubated at 50°C for 20 min, with mixing by vortexing at 10 min intervals. After inactivation of Proteinase K at 75°C, the lysate was treated with 0.5 μl of RQ1RNase-free DNase (Promega) at 37°C for 30 min. DNase was then inactivated with 1.0 μl of DNase stop solution (Promega) at 65°C for 10 min. For reverse transcription, 0.4 μl of DEPC-treated water, 0.8 μl of 10× first strand buffer (Applied Biosystems), 0.8 μl of 100 mM dNTP, 2 μl of 10× random primers, 0.6 μl of RNase Inhibitor, and 1 μl of Multiscribe reverse transcriptase (Applied Biosystems) were added. The reaction solution was incubated at 25°C for 10 min, 37°C for 2 h and 85°C for 5 min. RT-PCR for *cyclophilin*, *gnrh*, *gfap* (glial fibrillary acidic protein), *gpr54*, other target genes expression was carried out in a Mastercycler (Eppendorf, Germany) in a 10-μl reaction volume comprising 1× PCR Gold Buffer (Applied Biosystems), 2.5 mM MgCl_2_ (Applied Biosystems), 0.2 mM each dNTP (iDNA, Singapore), 0.25 U of AmpliTaq Gold A (Applied Biosystems), 0.25 μM each forward and reverse primers (Table S1 in Supplementary Material), and 1 μl of cDNA sample. PCR cycles were carried out under the following conditions: 95°C for 10 min, 40 cycles of 95°C for 30 s, 60°C for 30 s, and 72°C for 30 s, with a final extension phase of 72°C for 5 min. PCR products were electrophoresed on a 2.5% agarose gel, stained with ethidium bromide and visualized under an electronic UV transilluminator (ATTO, *Bio-Instrument*, Tokyo, Japan).

### Statistical Analysis

All data are expressed as a mean ± SEM for each group. Statistical analysis was carried out using PASW Statistics 18 (IBM SPSS Statistics; IBM, Chicago, IL, USA) software. The analysis of live cell imaging data was done using two-way repeated measure analysis of variance (ANOVA). The analysis of gene expression was done using *t*-test. Difference were considered significant when *p* < 0.05.

## Results

### Migration of Embryonic EGFP–GnRH Neurons in the Brain

**Figure 1:**
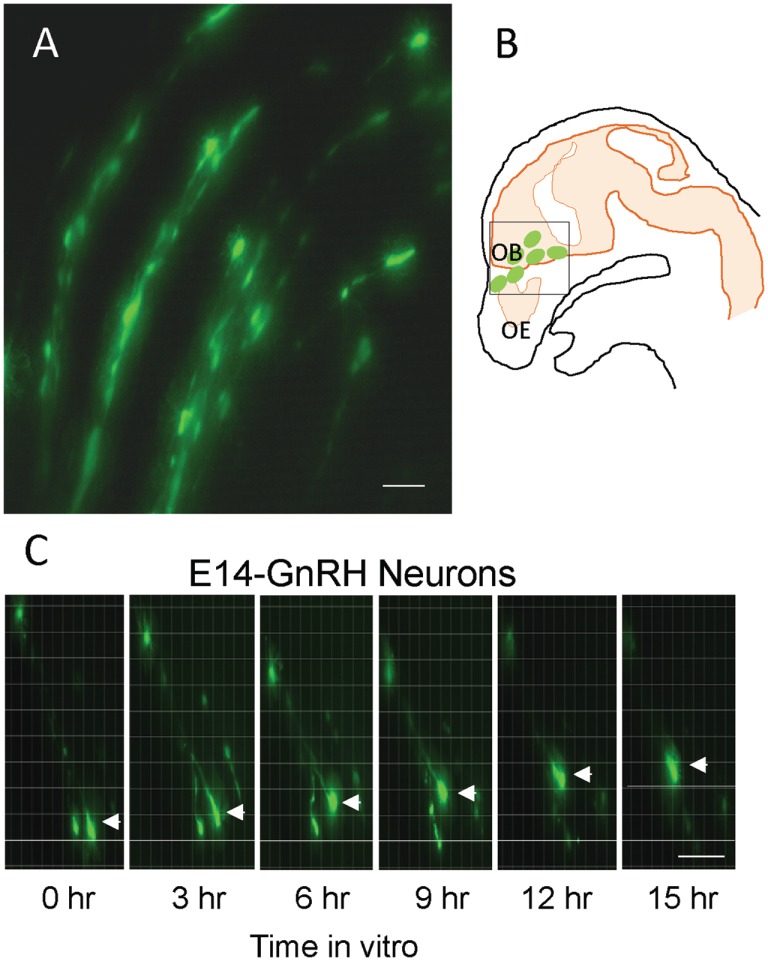
**Enhanced green fluorescent protein (EGFP)–gonadotropin-releasing hormone (GnRH) neurons on brain tissue in embryonic day 14 (E14) (A)**. Illustration of E14 rat sagittal head showing EGFP–GnRH neurons throughout olfactory bulb and ventral forebrain region and highlighted the area for live cell imaging **(B)**. Images of migrating EGFP–GnRH neurons in brain tissue culture captured by live cell imaging system during 15 h *in vitro* **(C)**. Scale bar = 50 μm in **(A,C)**.

**Figure 2:**
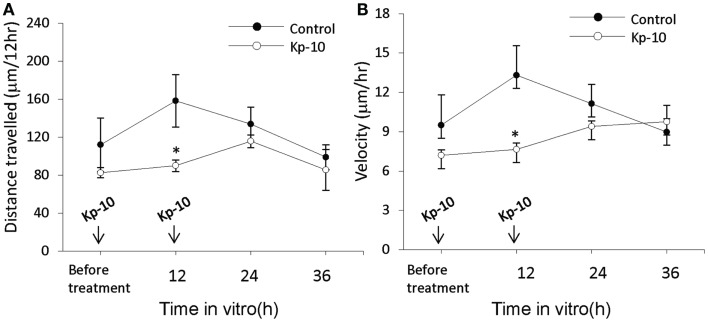
**Time-lapse imaging analysis of the enhanced green fluorescent protein (EGFP)–gonadotropin-releasing hormone (GnRH) neurons in brain tissue cultures from embryonic day 14**. Total distance traveled (μm/12 h) **(A)** and average rate of movement (μm/h) **(B)** [control, *n* = 7−10 cells/time point; Kisspeptin-10 (Kp-10), *n* = 7−10 cells/time point] determined across the 36 h culture duration. Data are represented by the mean ± SEM for each group. *p < 0.05 compared to control group.

The EGFP–GnRH neurons were identified by their fusiform morphology in the brain slice preparation of E14 rat brains (Figures 1A,B) and maintained *in vitro* for up to 36 h for time-lapse image analysis. The EGFP–GnRH neurons at E14 stage exhibited changes in their position (=were migrating) at different rates of motility within the brain slice during the 36 h of culture condition (Figure 1C). Time-lapse image analysis of the EGFP–GnRH neurons from E14 (*n* = 6–13 cells/time point) were expressed as the total distance traveled (μm/12 h) and the average rate of movement (μm/h) at 12 h interval for 36 h duration (Figure 2). The distance traveled by kisspeptin-10 exposed EGFP–GnRH neurons was significantly decreased at the 12-h culture time point [control, 158.2 ± 27.5 μm, kisspeptin-10, 90.0 ± 6.12 μm; *t*(11) = 2.91; *p* < 0.05]. After 24 and 36 h of the exposure to kisspeptin, the distance traveled by EGFP–GnRH cells was same as the control EGFP–GnRH neurons (Figure 2A). The average rate of movement of EGFP–GnRH cells was decreased in the 12-h culture time point for the exposure to kisspeptin [control 13.3 ± 2.3 μm/h, kisspeptin-10 exposed 7.6 ± 0.46 μm/h; *t*(11) = 2.43; *p* < 0.05]. The EGFP–GnRH neurons from the kisspeptin-10 exposed brain slice culture exhibited reduce motility compared to the control group maintained *in vitro* for 12 h. But after 24 h and 36 h of the exposure to kisspeptin, the average rate of movement of the EGFP–GnRH cells was same as the control EGFP–GnRH neurons (Figure 2B).

### GPR54 Gene Transcripts in Embryonic EGFP–GnRH Neurons in the Brain

**Figure 3:**
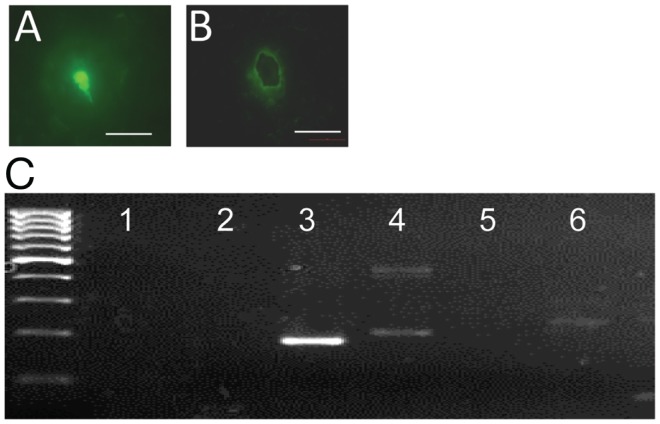
**G-protein-coupled receptor 54 (GPR54) mRNA expression in laser-captured enhanced green fluorescent protein (EGFP)–gonadotropin-releasing hormone (GnRH) neurons in brain tissue cultures from embryonic day 14**. **(A)** Photographs of EGFP–GnRH neurons in brain tissue culture from embryonic before and **(B)** after laser capture. Scale bar = 50 μm. **(C)** Composite gel showing amplicons of GnRH (105 bp, lane 2), EGFP (179 bp, lane 3), cyclophiline (199 bp, lane 4), GnRH receptor (100 bp, lane 5), GPR54 (235 bp, lane 6) in laser-captured microdissected GnRH neurons, and non-template controls (NTC, lane 1) Marker, DNA 100-bp ladder.

To determine whether *gpr54* gene transcripts are detected in embryonic EGFP–GnRH neurons, the expression of *gpr54* gene in embryonic EGFP–GnRH neurons captured by laser dissection was measured using real-time PCR (Figures 3A,B). *Gpr54* gene and *egfp* gene transcripts were detected in the laser-captured EGFP–GnRH neurons of E14 rat brains (Figure 3C). However, *gnrh* and *gnrh receptor* gene transcripts were not seen at this developmental stage of EGFP–GnRH neurons.

### Effect of Kisspeptin on Apoptosis, Cell Proliferation, and Cytoskeleton-Related Genes Expression in Embryonic EGFP–GnRH Neurons

**Figure 4:**
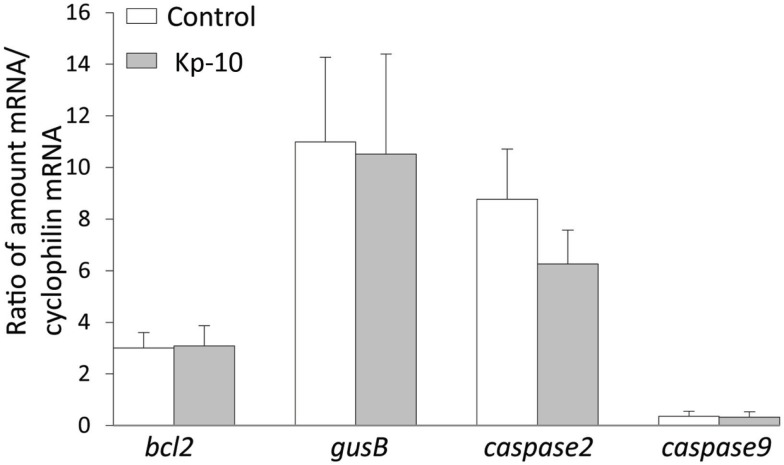
**Effects of kisspeptin-10 treatment on apoptosis-related genes expression in laser-captured EGFP–GnRH in brain tissue culture from embryonic (E) day 14**. Data are presented as the mean ± SEM [control: *N* = 20−25 cells/animals (*n* = 6), kisspeptin-10 (Kp-10): *N* = 20−25 cells/animals (*n* = 6)]. bcl2, B-cell lymphoma 2; gusB, glucuronidase; Beta, caspase 2/9, cysteine-aspartic proteases or cysteine-dependent aspartate-directed proteases 2/9.

**Figure 5:**
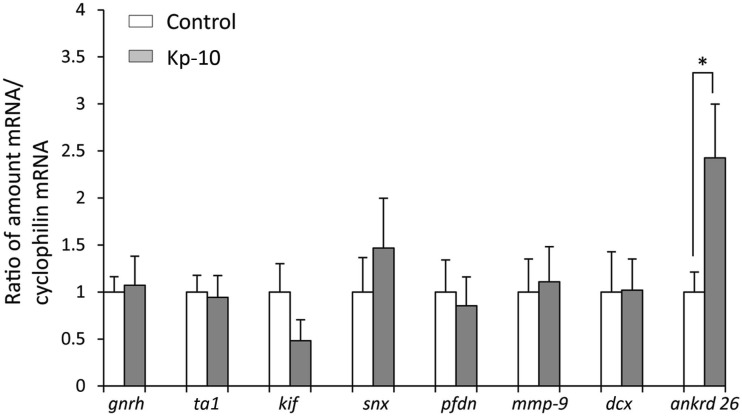
**Effects of kisspeptin-10 treatment on cell proliferation and cytoskeleton-related genes expression in laser-captured EGFP–GnRH in brain tissue culture from embryonic day 14**. Data are presented as the mean ± SEM [control, *N* = 20−25 cells/animals (*n* = 6); kisspeptin (Kp-10), *N* = 20−25 cells/animals (*n* = 6)]. *p < 0.05 compared between Kisspeptin-10 (1 nM) and control group. gnrh, gonadotropin-releasing hormone; *ta1*, tubulin a1; kif, kinetin family; snx, sorting nexin18; pfdn, prefolding 6; mmp-9, matrix metalloproteinase; *dcx*, doublecortin; ankrd 26, ankyrin repeat domain-containing protein 26.

To determine whether kisspeptin alter apoptosis, cell proliferation, and cytoskeleton in migrating embryonic EGFP–GnRH neurons, levels of apoptosis, cell proliferation, and cytoskeleton-related gene expression was determined in migrating embryonic EGFP–GnRH neurons after 12 h exposure to kisspeptin. Apoptosis-related genes, *B-cell lymphoma* (*bcl*) *2*, *glucuronidase*, *Beta* (*gusB*), *cysteine-aspartic proteases, or cysteine-dependent aspartate-directed proteases* (*caspase*) *2* and *9* were selected to measure the mRNA levels in laser-captured GnRH neurons from E14 brain slice culture. There was no difference in the expression of these genes between control and exposure to kisspeptin (Figure 4). On the other hand, cell proliferation and cytoskeleton-related genes, *gnrh*, *tubulin* α*1* (*tα1*), *prefolding 6* (*pfdn*), *kinetin family* (*kif*), *sorting nexin18* (*snx*) *matrix metalloproteinase* (*mmp*)*-9*, *doublecortin* (*dcx*), and *ankyrin repeat domain-containing protein* (*ankrd*) *26* genes were selected to measure their mRNA levels in laser-captured GnRH neurons from E14 brain slice culture. There was no difference in the level of expression of these genes between control and kisspeptin exposed neurons (Figure 5). However, the expression of *ankrd*26 mRNA was upregulated by exposure to kisspeptin in laser-captured GnRH neurons from E14 brain slice culture [control: 1.0 ± 0.21, kisspeptin: 2.41 ± 0.57; *t*(11) = −2.34, *p* < 0.05] (Figure 5).

## Discussion

In this study, real-time imaging and capturing of live EGFP–GnRH neurons from E14 brain slice cultures facilitated time-lapse analysis and characterization of embryonic GnRH neuronal movement following exposure to kisspeptin. Kisspeptin decreased GnRH neuronal movement in brain slices and increase *ankrd26* gene expression in laser-captured GnRH neurons but had no effect on apoptosis and cell proliferation-related genes. These data suggest that exposure to kisspeptin may inhibit GnRH neuronal movement through novel intracellular *ankrd26* signaling.

### Effects of Kisspeptin on GnRH Neuronal Movement and Gene Expression in the Brain

Time-lapse image analysis of rat embryonic E14–GnRH neurons between the nasal and brain compartments showed movement of GnRH neurons at a velocity of 9–13 μm/h in brain slice cultures similar to E13 GnRH neurons in embryonic mouse nasal explants culture migrating at a velocity of 12–13 μm/h (38). On the other hand, our recent time-lapse imaging study of rat GnRH neurons on postnatal day 0 and day 5 showed movement of GnRH neurons at a velocity of 3–5 μm/h (39). This suggests that rat embryonic GnRH neurons have high motility and, therefore, may be a good model to determine intracellular signaling for cellular movement. Exposure to kisspeptin reduced the distance traveled and velocity of embryonic GnRH neurons after 12 h but not after 24 h of treatment. Percentage of GPR54-expressing GnRH neurons dramatically is increased during E14.5–16.5 in mice (21). This suggests kisspeptin–GPR54 signaling directly exerts an acute but transient effect on the movement of GnRH neurons during early development. Several inhibitory signaling such as GABA in GnRH migration are identified (14, 40), kisspeptin–GPR54 singling may involve in slow down or halt GnRH migration for fine-tuning of GnRH development. During embryonic stage E14–18, multiple signal transduction pathways are engaged to modulate GnRH neuronal movement (38, 41). Although the mechanism of kisspeptin–GPR54 in suppressing metastasis is still incompletely characterized, previous research has shown kisspeptin-10 as an anti-metastatic agent through complex cellular signaling including loss of intracellular adhesion, stromal invasion, and attachment at distant sites in several types of cancer cells (30, 42–49). A recent study reported that kisspeptin-10 affects invasion *via* modulation of MMP 9 activity in breast cancer cells (50). However, kisspeptin-10 does not affect the expression of MMP9 gene expression in migrating embryonic GnRH neurons in brain slice cultures. Therefore, the effects of kisspeptin-10 on migrating GnRH neurons might involve a different mechanism. A metastasis-associated protein family (MTA) has been identified to promote tumor cell invasion and metastasis in human brain glioma (49). Recent evidence proposes that GPR54 is expressed in hypoxic areas and endothelial cells of tumor blood vessels of glioblastoma (51). Thus, it is possible that kisspeptin-10 may be involved in neuronal or glia specific cellular mechanism of metastasis *via* a novel molecule such as MTA. In our study, kisspeptin-10 had no effect on apoptosis genes (*bcl 2*, *gusB*, *caspase 2 and 9*) and cell proliferation and cytoskeleton related genes (*tα1, pfdn6, kif, snx, mmp-9 and dcx*) in migrating embryonic GnRH neurons. Kisspeptin-10 exposure may affect factors related migrating during developmental stage but not apoptosis.

### Effect of Kisspeptin-10 Treatment on *Ankrd 26* Signaling

Kisspeptin-10 treatment increased *ankrd* 26 mRNA levels and inhibited the movement/migration of EGFP–GnRH neurons in embryonic brain slice cultures. In our previous study, we identified *ANKRD* protein coding gene expression in laser capture GnRH neurons from E14 and postnatal day 5 (P5) brains through a microarray analysis (52). The levels of *ankrd* 26 mRNA was higher in p5 than, the highly motile and migrating E14 stage GnRH neurons. Therefore, cellular *ankrd* 26 signaling may be involved in the anti-migration process in neurons. Ankyrin repeat is a key amino acid motif as a scaffold for protein–protein interactions (53, 54), thus ankyrin repeat mediated protein–protein interactions may ultimately help to unravel complex cell signaling mechanisms related to the migration process in neurons. Interestingly, *ankrd* 26 gene belongs to the POTE family of genes that are highly expressed in the prostate, ovary, testis, and placenta of cancer patients (55), which also express kisspeptin and GPR54. This suggests that *ankrd* 26 gene expression in reproductive organs may be regulated by kisspeption-GPR54 signaling. Retinoic acid receptor responder (RARRES1) is a putative tumor suppressor (56) gene that suppress invasion and colony-forming ability of prostate cancer cells (57). Since, knockdown of *RARRES1* gene upregulates *ANKRD* 26 protein (56), it would be important to see if kisspeptin regulates *ankrd* 26 through *RARRES1*-mediated pathway in neuronal migration. *ankrd* 26 gene expression is reported in the hypothalamus area, and it is suggested to involve in obesity and gigantism (58). A little knows about the potential physiological functions of *ankrd* 26 in the brain during embryonic stage. Further studies are needed to understand the direct action of kisspeptin on *ankrd* 26 gene expression and the functions in migrating GnRH neurons.

## Conclusion

In this study, exposure to kisspeptin reduced the movement of embryonic EGFP–GnRH neurons in brain slice culture. We found that there was no change in the expression of cell proliferation, cytoskeleton and apoptosis-related genes. However, *ankrd* 26 gene in migrating embryonic EGFP–GnRH neurons was upregulated by exposure to kisspeptin. These studies support that *ankrd* 26 signaling plays an unidentified role to regulate neuronal movement mediated by kisspeptin–GPR54 signaling. Therefore, *ankrd* 26 could be a specific target for elucidating the mechanism for suppressing one of pathway in neuronal metastasis by kisspeptin–GPR54 signaling. Although kisspeptin–GPR54 system in the embryonic brain has only recently been studied, our studies suggest that kisspeptin–GPR54 exerts a part of potential function on embryonic GnRH development.

## Author Contributions

Study concept and design: IP and TS; acquisition of data: TS and WL; analysis and interpretation of data: IP, WL, and TS; drafting of the manuscript: WL and TS; statistical analysis: WL and TS; critical revision of manuscript: IP and AK; obtained funding: IP, TS, WL, and AK.

## Conflict of Interest Statement

The authors declare that the research was conducted in the absence of any commercial or financial relationships that could be construed as a potential conflict of interest.
