# Supplementary material for: Kisspeptin Activates Ankrd 26 Gene Expression in Migrating Embryonic GnRH Neurons
Source: Front Endocrinol (Lausanne). 2016 Mar 1;7:15. doi: 10.3389/fendo.2016.00015 (PMC4771921; doi:10.3389/fendo.2016.00015)
Supplement: Supplementary file 1 [file Table_1.docx]

| **Supplemental table 1** |  |  |  |
| --- | --- | --- | --- |
| **Gene** | **primers (5`-3`)** | **Product size(bp)** | **Accession number** |
| Gonadotropin-releasing hormone | AGC TCT GGA ACG TCT GAT TGA AG | 105 | NM_012767 |
| (gnrh) | TGG ATC TCA GCG TCA ATG TCA |  |  |
|  |  |  |  |
| cyclophylin | ATA ATG GCA CTG GTG GCA AG | 199 | NM_017101 |
|  | CAT GCC TTC TTT CAC CTT CC |  |  |
|  |  |  |  |
| Gonadotropin-releasing hormone  receptor (gnrh-r) | CCCACGCAAACTACAGCTGAA | 100 | NM_031038. |
|  | GATGACAAAGGAGGTGGCAAA |  |  |
|  |  |  |  |
| G-protein coupled receptor 54(gpr54) | GCCGCAGAGGCGACGTTGGG | 235 | NM_053244 |
|  | AGAAATTGGTGACGGTCTGC |  |  |
|  |  |  |  |
| Caspase 2 | ACTGGAATGTCAGCTCGCAATGGT | 85 | NM_012922 |
|  | TCCTGACTTCGTATTTCAGGGCCA |  |  |
|  |  |  |  |
| Caspase 9 | TGAACATCTTCAATGGGACCGGCT | 144 | NM_031632 |
|  | AGTCACTGTCGAAGGCCTTGTCTT |  |  |
|  |  |  |  |
| B-cell leukemia/lymphoma 2(bcl2) | TTGTGGCCTTCTTTGAGTTCGGTG | 93 | NM_016993 |
|  | TCATCCACAGAGCGATGTTGTCCA |  |  |
|  |  |  |  |
| glucuronidase, beta(gusB) | ACGCTGATCGCCCACACCAA | 83 | NM_017015 |
|  | CCCATGTCTGCGTCATATCTGGTA |  |  |
|  |  |  |  |
| Prefoldin 6(pfdn) | ATGTCAGGGAGGCAGAAGCTTGAA | 114 | NM_212506 |
|  | AAGCACGGGTCCCAGAAGCTTAAA |  |  |
|  |  |  |  |
| Matrix metallopeptidase 9 (mmp9) | AGAATCTCTACACGGAGCATGGCA | 98 | NM_031055 |
|  | ACCTTTAGTGGTGCAGGCAGAGTA |  |  |
|  |  |  |  |
| Kinesin family member 13A(kif) | ACCGAAGAGAGCTGGAACTGAACA | 101 | XM_217893 |
|  | TCTTTCTCCCTGCTTGGTGTTGGA |  |  |
|  |  |  |  |
| Tubulin, alpha 1A (tα1) | AGATGCCAAGCGACAAGACCATTG | 145 | NM_022298 |
|  | TAGGTGCCAGTGCGAACTTCATCA |  |  |
|  |  |  |  |
| Sorting nexin 18 (snx) | GCCAACTTCCCAGACATCATCCAT | 86 | NM_001107652 |
|  | ATCTTCCCTTCTTCCACGTGTCGT |  |  |
|  |  |  |  |
| Doublecortin (dcx) | TCGTAGTTTTGATGCGTTGC | 146 | NM_053379 |
|  | GCTTTCCCCTTCTTCCAGTT |  |  |
|  |  |  |  |
| Ankyrin repeat domain 26 | GCGCTCCTGTCCTGGTTTT | 105 | XM_224750 |
| (ankrd26) | AGGCCGCTCTTTGTAATCGA |  |  |
|  |  |  |  |
| Glial fibrillary acidic protein | TTCCTGTACAGACTTTCTCCAACCT | 100 | NM_001131020 |
| (gfap) | GTCTTTACCACGATGTTCCTCTTGA |  |  |
|  |  |  |  |
| Enhanced green fluorescent protein | ACTTCAAGATCCGCCACAAC | 179 | CVU55761 |
| (egfp) | GAACTCCAGCAGGACCATGT |  |  |
|  |  |  |  |
